# Supplementary material for: Breaking Barriers to Rapid Whole Genome Sequencing in Pediatrics: Michigan’s Project Baby Deer
Source: Children (Basel). 2023 Jan 4;10(1):106. doi: 10.3390/children10010106 (PMC9857227; doi:10.3390/children10010106)
Supplement: Supplementary file 1 [file children-10-00106-s001.zip › children-2062723-supplementary.pdf]

Supplementary Figure S1. PBD Change in Management Assessment.

project baby deer

Project Baby Deer Change in Management Assessment

Patient Name:______________________________ CS Number:______________________________

Clinical Utility of Genomic Testing (select one):				
1 – Not useful at all	2 – Not very useful	3 – Neutral	4 – Useful	5 – Very Useful

Genomic findings… (select all that apply):
◻ allowed avoidance of complications.<br>◻ enabled targeted treatment that may improve long-term outcomes.<br>◻ enabled improved communication of outcomes/expectations/prognosis with families.<br>◻ increased stress for family.<br>◻ increased confusion for family.<br>◻ increased confusion among clinical staff.<br>◻ resulted in a diagnosis not fully understood at this time.

Change in Clinical Management (select all that apply):		
Surgical Intervention	◻ Added<br>◻ Changed<br>◻ Removed	Explanation:________________________
Medication	◻ Added<br>◻ Changed<br>◻ Removed	
Hospital Days	◻ Added number of days:____________<br>◻ Avoided number of days:____________	
Transplant	◻ Added<br>◻ Withdrawn	
Dietary	◻ Added<br>◻ Removed	
Transfer to specialist center	◻ Initiated<br>◻ Cancelled	
Other	◻ Added<br>◻ Changed<br>◻ Removed	

Other Management Changes (select all that apply):		
Specialty Services	◻ Added<br>◻ No longer required	Explanation:________________________
Imaging	◻ Added<br>◻ Cancelled	
Testing	◻ Added<br>◻ Cancelled	
Screening for additional comorbidities	◻ Added<br>◻ Removed	
Condition specific management	◻ Added<br>◻ Removed	
Condition specific supportive care	◻ Added<br>◻ Removed	
Other	◻ Added<br>◻ Changed<br>◻ Removed	

Genetic Tests Avoided (select all that apply):			
◻ Karyotype	◻ FISH	◻ Microarray	◻ WES
◻ Cytogenetic testing	◻ Metabolic testing	◻ SMA testing	◻ Prader Willi
◻ Next generation sequencing panel:		Other:	

Supplementary Table S1. Patient Demographics.

**Race**	**Total (n=89)**
African American	11 (12%)
Asian	4 (4%)
Caucasian	64 (72%)
Other	5 (6%)
Unknown	5 (6%)
**Ethnicity**	
Hispanic	10 (11%)
Non-Hispanic	72 (81%)
Other	6 (7%)
Unknown	1 (1%)
**Sex**	
Female	32 (36%)
Male	57 (64%)
